# The Impact of Coping Styles and Gender on Situational Coping: An Ecological Momentary Assessment Study With the mHealth Application TrackYourStress

**DOI:** 10.3389/fpsyg.2022.913125

**Published:** 2022-06-20

**Authors:** Teresa O’Rourke, Carsten Vogel, Dennis John, Rüdiger Pryss, Johannes Schobel, Fabian Haug, Julian Haug, Christoph Pieh, Urs M. Nater, Anja C. Feneberg, Manfred Reichert, Thomas Probst

**Affiliations:** ^1^Department of Psychosomatic Medicine and Psychotherapy, University for Continuing Education Krems, Krems an der Donau, Austria; ^2^Institute of Clinical Epidemiology and Biometry, University of Würzburg, Würzburg, Germany; ^3^Lutheran University of Applied Sciences, Nuremberg, Germany; ^4^DigiHealth Institute, Neu-Ulm University of Applied Sciences, Neu-Ulm, Germany; ^5^Faculty of Engineering, Computer Sciences and Psychology, Institute for Databases and Information Systems, University of Ulm, Ulm, Germany; ^6^Department of Clinical and Health Psychology, University of Vienna, Vienna, Austria

**Keywords:** coping, stress, Ecological Momentary Assessment (EMA), mHealth, mobile application

## Abstract

The aim of this study was to investigate the impact of different coping styles on situational coping in everyday life situations and gender differences. An ecological momentary assessment study with the mobile health app TrackYourStress was conducted with 113 participants. The coping styles *Positive Thinking*, *Active Stress Coping*, *Social Support*, *Support in Faith*, and *Alcohol and Cigarette Consumption* of the Stress and Coping Inventory were measured at baseline. Situational coping was assessed by the question “How well can you cope with your momentary stress level” over 4 weeks. Multilevel models were conducted to test the effects of the coping styles on situational coping. Additionally, gender differences were evaluated. *Positive Thinking* (*p* = 0.03) and *Active Stress Coping* (*p* = 0.04) had significant positive impacts on situational coping in the total sample. For women, *Social Support* had a significant positive effect on situational coping (*p* = 0.046). For men, *Active Stress Coping* had a significant positive effect on situational coping (*p* = 0.001). Women had higher scores on the SCI scale *Social Support* than men (*p* = 0.007). These results suggest that different coping styles could be more effective in daily life for women than for men. Taking this into account, interventions tailored to users’ coping styles might lead to better coping outcomes than generalized interventions.

## Introduction

Coping is defined as the ability to manage a situation that an individual perceives as threatening, stressful, or burdensome (Carver, 2011). Adaptive coping is necessary on a daily basis to alleviate stress consequences of daily life stress experiences, and to prevent negative health consequences of severe and chronic stress (Cohen et al., 2007, 2016). One of the most established models of coping is the transactional stress model by Lazarus and Folkman (1984). According to this model, stress responses to a situation are influenced by the cognitive appraisals of the situation as well as personal strategies to cope with stressful situations.

### Coping Styles

Such coping strategies are targeted either to actively resolve the stressful situation (problem-oriented), or to reduce the emotional consequences of this situation (emotion-oriented; Lazarus and Folkman, 1984). They can be influenced by personal factors such as personal goals or beliefs and situational factors such as the novelty or predictability of a stressful situation. In addition to this classification, coping strategies can also be divided into approach and avoidance strategies (Taylor and Stanton, 2007). An approach strategy is, for example, the active removal of a stressor, while the withdrawal from a situation that is perceived as stressful is an avoidance strategy. Avoidance coping strategies can lead to an increased subjective stress reaction (Frazier et al., 2005) while approach strategies are associated with better coping results, such as better psychological adjustment (Roesch and Weiner, 2001). Coping strategies can furthermore be described as more adaptive or maladaptive. This distinction is often made, but whether something is adaptive or not depends on the chosen outcome, the time frame, and the context, which makes this distinction complicated (Skinner et al., 2003). Skinner et al. (2003) nevertheless argue, that while specific coping styles can be both adaptive and maladaptive, depending on situational factors, a distinction between adaptive and maladaptive ways of coping can be made based on long-term developmental consequences, subjective experiences, and situational qualities. They describe adaptive ways of coping as generally more organized, flexible, and constructive, and maladaptive ways of coping as more rigid, disorganized, or derogatory. Examples for adaptive coping strategies are active stress coping, positive thinking, social support, support in faith, and an example for a maladaptive coping strategy is alcohol and cigarette consumption (Satow, 2012). Some coping strategies, such as avoidance, can be adaptive for some time but maladaptive in the long-run (Seiffge-Krenke and Klessinger, 2000). Adaptive coping strategies can have protective effects on physical and mental health (McPherson, 2003). A lack of adaptive coping strategies has been associated with the experience of chronic stress (Repetti et al., 2002) and may be a risk or maintaining factor of various mental disorders.

### Interindividual and Intraindividual Differences in Coping

Coping can vary between individuals as well as within a person (Schneiderman et al., 2005). For example, interindividual differences have been shown for gender. Results of a meta-analysis on gender differences in coping styles suggest that women are generally more likely than men to use coping strategies such as active coping, seeking social support or using religion to cope with stressful situations (Tamres et al., 2002). Besides interindividual differences, intraindividual differences should also be taken into account when researching coping. For example, coping might differ within an individual depending on the frequency and intensity of stressors over time (Schneiderman et al., 2005). In cross-sectional and laboratory studies, however, such intraindividual processes can only be insufficiently considered, as they often rely on retrospective assessments (Shiffman et al., 2008).

### Ecological Momentary Assessment

Contrary to laboratory studies, a method of data acquisition that enables the generalization of results to real-world contexts is Ecological Momentary Assessment (EMA), which is defined by the assessment of data in real-world environments, in real time, and over multiple measurement time-points (Stone and Shiffman, 1994). The collection of data in real-world environments is beneficial, because behaviors and experiences are assessed in the context they typically occur in and individual context variables such as time of day can be taken into account (Shiffman et al., 2008). Another advantage of this type of investigation is that it enables the measurement of coping in real time, so that any distortions caused by retrospective assessments can be avoided. For example, people tend to be more likely to remember situations that are more relevant to them personally, happened more recently, or are unusual, which can bias retrospective reports (Trull and Ebner-Priemer, 2009). By including repeated measures, within-subject changes in behavior and experience over time can be investigated (Shiffman et al., 2008).

Stone and Neale (1984) conducted one of the earliest EMA-related studies regarding coping to create an assessment tool for daily coping. Within a bigger longitudinal study, they sent out questionnaires to 120 married couples, which filled these out over a period of 21 days. Among other things, they found that men used direct action as a coping strategy more often, whereas women used other strategies such as seeking social support or religion more often (Stone and Neale, 1984). More recently, with the increasing use of smartphones and other electronic devices in the general population, EMA designs have become more widely applied in various areas of psychological research (Aan Het Rot et al., 2012; Bos et al., 2015). With the advance of digitalization, smartphone-based mobile health (mHealth) apps, which focus on medical and public health practice as well as health promotion (Kazdin, 2017), have become popular. They are a low-threshold option of mental health support, as they require little effort to engage with, can be used anywhere and have the potential to reach large numbers of people (WHO Global Observatory for eHealth, 2011). Such mHealth apps facilitate the integration of questionnaires into daily life, which enables the assessment of dynamic coping processes over time with EMA designs in contrast to retrospective assessments, which cannot take such dynamic changes into account. EMA therefore provides a convenient strategy for collecting data with such mHealth apps.

### Objectives

The main objective of this study was to investigate the impact of five coping styles (positive thinking, active stress coping, social support, support in faith, and alcohol and cigarette consumption) on situational coping in everyday life and which coping styles affect situational coping in women and men, respectively. Another aim was to determine whether men and women differ in their coping styles and whether gender moderates the effects of the coping styles on situational coping.

### The Present Study

Based on this theoretical background, this study addressed the following research questions (RQ):

– RQ 1. Which coping styles are associated with situational coping in the total sample? For this RQ, we hypothesized that the coping styles would differ in their impact on situational coping because previous research (Seiffge-Krenke and Klessinger, 2000; Satow, 2012) indicates that coping styles are not equally effective at resolving stress reactions.
– RQ 1a. Which coping styles are associated with situational coping for women? For this RQ, we hypothesized that the coping styles would differ in their impact on situational coping for women.
– RQ 1b. Which coping styles are associated with situational coping for men? For this RQ, we hypothesized that the coping styles would differ in their impact on situational coping for men.
– RQ 1c. Does gender moderate the effect of the coping styles on situational coping? For this RQ, we hypothesized that the effect of the coping styles on situational coping would be moderated by gender.
– RQ 2. Are there differences in the coping styles between women and men? For this RQ, we hypothesized that gender differences in coping styles would emerge because previous research has found gender-differences in coping, for example, in regard to seeking social support or religion (Satow, 2012).

## Materials and Methods

### Study Design

In the course of this study, data were analyzed that were collected by three student researchers in an EMA study with the Crowdsensing Platform and App TrackYourStress from July 2018 to January 2019 (Pryss et al., 2019a,b). The study was conducted in accordance with the Declaration of Helsinki. After filling out the baseline questionnaire on the TrackYourStress app, participants rated their subjective stress experience and coping over a course of 4 weeks. To monitor stress and coping over the 4 weeks, the participants were instructed to fill out the app’s daily questionnaire (see Table 1) on their own mobile phones. There were two ways how participants could answer the daily questionnaire. First, the participants could set notifications to be reminded at self-chosen times in their regular daily life to fill in the daily questionnaire including the question on situational coping. Second, they could self-initiate to fill in the daily questionnaire including the question on situational coping.

**Table 1:** Daily questionnaire.

1. How high is your momentary stress level?
2. How well can you cope with your momentary stress level?
3. How strongly are you experiencing your momentary stress level as negative/impairing?
4. How strongly are you experiencing your momentary stress level as positive/beneficial?
5. What stresses you at the moment?
6. How is your mood right now?
7. How is your arousal right now?
8. How important is the current situation for you personally?
9. How would you assess your resources to manage the currently experienced situation?

### Sample and Procedure

Three students from the FOM University of Applied Sciences in Augsburg and Munich recruited the participants in this study. The sample was a convenience sample recruited at these universities and in the circles of acquaintances and colleagues of the three student recruiters. To partake in this study, participants had to be at least 18 years old, own a smartphone with either Android or iOS system software, and agree to use the TrackYourStress app for 4 weeks (informed consent). Possible participants were invited per e-mail and could register for study participation after giving informed consent. Participants did not receive any kind of compensation for partaking in the study.

The mobile application was downloaded *via* private installation routines on the participants’ devices. Each participant then completed the registration with a password-protected user profile. After this procedure, the participants completed the baseline assessment by filling out a registration questionnaire on the TrackYourStress app including the coping scale of the stress and coping inventory (SCI; Satow, 2012; see below). The participants were then asked to fill out the questions of the daily questionnaire (see below) over a course of 4 weeks each. The assessments were not event-based and could be made at random points of time.

### Materials

#### TrackYourStress

TrackYourStress is a mHealth crowdsensing platform with smartphone apps for iOS and Android. TrackYourStress was developed by researchers from the Danube University Krems, the University of Ulm, the University of Würzburg, and the Lutheran University of Applied Sciences in Nuremberg to measure subjective stress experiences and coping in everyday life. The app will soon be freely available in the App Stores for both iOS and Android. The app offers users the opportunity to systematically measure their individual fluctuations in stress levels and find out how these are related to various events in their daily lives. TrackYourStress contains several established questionnaires that have been shown to have good psychometric qualities and are as short as possible, so that participants remain motivated to complete them repeatedly over a longer period of time. It also includes a feedback function, which consists of a visualization of the users’ scores on the different questionnaires and is designed to facilitate health promotion.

#### Registration Questionnaire

In a registration questionnaire, in addition to basic demographic variables, the individual stress reactivity was assessed with the Perceived Stress Reactivity Scale (PSRS; Schlotz et al., 2011), the stress coping of the last month with the Stress and Coping Inventory (SCI; Satow, 2012), and the stress level of the last 7 days with the 4-item short version of the Perceived Stress Scale (PSS-4; Cohen et al., 1983). In the latest version of TrackYourStress, a personality questionnaire is included in the registration questionnaire as well.

#### Daily Questionnaire

The app’s daily questionnaire (Table 1) consists of various questions on situational stress levels and situational coping. These questions were designed to address the main concepts of the transactional stress model (Lazarus and Folkman, 1984).

#### Weekly and Monthly Questionnaires

The app also contains a weekly questionnaire, which assesses the stress level of the last 7 days and a monthly questionnaire that again covers coping styles. The latter two questionnaires, however, are not relevant for this paper, which focuses on the SCI coping scales of the registration questionnaire and the daily questionnaires’ item on situational coping.

#### Feedback in TrackYourStress

Additionally, in the latest version of TrackYourStress, users can view their data history and receive personalized feedback on their entries, designed to facilitate health promotion. TrackYourStress can be used as a self-management tool to identify individual stress fluctuations. In the TYS pilot study on measurement reactivity (Pryss et al., 2019b), it was ascertained that using the TYS app over a prolonged period of time itself does not influence the self-reports of the participants in the app.

### Measures

#### Coping styles

Coping styles in the Stress and Coping Inventory (SCI; Satow, 2012): To measure the participants’ individual coping styles, the SCI coping scales with 20 items are used in TrackYourStress. There are five coping scales in the SCI:

– *Positive Thinking* (*PT*; e.g., “I tell myself that stress and pressure also have their good sides.”),
– *Active Stress Coping* (*AS*; e.g., “I try to avoid stress in advance.”),
– *Social Support* (*SS*; e.g., “When I come under pressure, I have people who help me.”),
– *Support in Faith* (*SF;* e.g., “Under stress and pressure, I find stability in faith.”), and
– *Alcohol and Cigarette Consumption* (*AC*; e.g., “Under stress and pressure, I relax with a glass of wine or beer in the evening.”).

The scales each consist of four questions on a Likert scale from 1 (do not agree at all) to 4 (strongly agree). For most items, higher values represent a better fit with the respective coping strategy. Only in one item of the scale, *Alcohol and Cigarette Consumption* does a higher score indicate a worse fit with this particular coping strategy (“No matter how much stress I get, I would never turn to alcohol or cigarettes because of stress.”). This item has hence to be recoded. Higher scores on a scale level represent more use of PT, AS, SS, SF, AC as coping styles. While higher scores in four scales (PT, AS, SS, SF) represent adaptive coping, higher scores in the AC scale represent a more maladaptive coping. In our sample, the scales of the SCI showed the following internal consistencies (Cronbach alpha): *PT* (alpha = 0.70), *AS* (alpha = 0.77), *SS* (alpha = 0.90), *SF* (alpha = 0.77), and *AC* (alpha = 0.70). These values indicate acceptable to excellent reliabilities and are similar to the Cronbach alphas in the original sample [8]: *PT* (alpha = 0.74), *AS* (alpha = 0.74), *SS* (alpha = 0.88), *SF* (alpha = 0.78), *AC* (alpha = 0.75).

#### Situational Coping

In the daily questionnaire of TrackYourStress, the question on situational coping “How well can you cope with your momentary stress level?” was analyzed in the current paper. This question is answered on a slider/Visual Analogue Scale (VAS) from *not at all* to *very well* with values from 0 to 100. Higher values, therefore, indicate better situational coping.

### Statistical Analysis

SPSS v25 was used for all statistical analyses. All of the statistical tests were 2-tailed, and the significance level was set to *p* < 0.05. Addressing the nested data structure, linear multilevel models were conducted to test RQ 1, RQ 1a, RQ 1b, RQ 1c. The multilevel models had two levels: assessments as level 1 and individuals as level 2. The dependent variable was situational coping in all models. The five coping styles assessed with the SCI at baseline were entered as time-invariant covariates into one model in order to evaluate the effect of each SCI coping strategy on situational coping independent from the influence of the other SCI coping styles. To facilitate interpretation of the results, the scores of the SCI coping scales were z-standardized. In summary, three multilevel models were calculated, one for the total sample (RQ 1), one for women (RQ 1a), one for men (RQ 1b). For RQ 1c, the total sample was analyzed, but this model not only included the SCI coping scales as time-invariant covariates but also gender as another time-invariant covariate. The following main and interaction effects were examined in this model: gender, PT, AS, SS, SF, AC, gender x PT, gender x AS, gender x SS, gender x SF, gender x AC. All multilevel models were calculated with full maximum likelihood estimation. The intercept was allowed to vary in these models (random intercept models). When analyzing longitudinal data, multilevel models have more advantages over ANOVA or MANOVA (O’Connell and McCoach, 2004). To evaluate RQ 2, *t*-tests for independent samples were performed and Hedge’s *g* was calculated as effect size (0.2–0.5 = small effect; 0.5–0.8 = medium effect; >0.8 = large effect). Heteroscedasticity was tested with the Levene test.

## Results

A total of 113 participants (test users excluded) took part in the study. Of these, 65 were female and 47 were male. One participant did not state their gender. The participants’ ages ranged from 18 to 62 years with a mean age of *M* = 33.46 (*SD* = 11.36), whereby age was missing for two participants. These participants with missing values in age and gender were included in the further analyses of the hypotheses as well, if applicable. The participant with missing gender had to be excluded for RQ1a, b, c, and RQ2. During the study interval, the participants filled out the daily questionnaire (including the question on situational coping) a total of 2,227 times and a mean 1.03 (SD = 0.06) times per day. They reported mean stress levels from 0.00 to 75.70 (M = 33.56, SD = 16.44). Women reported significantly higher stress levels than men (*p* = 0.014). They reported situational coping scores ranging from 17.22 to 100 (*M* = 66.12, *SD* = 19.56).

Table 2 shows the zero-order correlations between the individual coping styles. All of the adaptive coping scales show significant positive correlations to each other. The maladaptive coping scale *Alcohol and Cigarette Consumption* does not correlate with any of the other coping scales.

**Table 2:** Correlations between coping styles.

	*N*	M	SD	1	2	3	4	5
1. PT	113	11.68	2.34	−				
2. AS	113	11.57	2.17	0.23^*^	−			
3. SS	113	13.51	2.66	0.27^**^	0.21^*^	−		
4. SF	113	6.90	2.30	0.25^**^	0.27^**^	0.24^*^	−	
5. AC	113	6.70	2.62	−0.10	−0.10	−0.12	0.02	−

* *p < 0.05*;

** *p < 0.001*.

*PT, positive thinking; AS, active stress coping; SS, social support; SF, support in faith; AC, alcohol and cigarette consumption*.

### Descriptive Statistics

Table 3 shows the results of the null model for the total sample, as well as for women and men separately.

**Table 3:** Results of the null model.

Parameter	Total sample	Women	Men
Intercept	66.78	62.46	73.47
Var. intercept	355.88	381.92	246.41
Var. residual	449.30	473.52	411.52
ICC	0.558	0.805	0.625

*ICC, Intraclass correlation*.

### Results for RQ 1

Table 4 shows the results of the linear multilevel model testing the effects of coping styles on situational coping for the total sample. The effect of the SCI scale *Positive Thinking* on situational coping was positive and significant (estimate = 4.11; *p* = 0.03). This means that higher scores in the coping strategy *Positive Thinking* were associated with higher situational coping in daily life. The effect of *Active Stress Coping* on situational coping was also positive and significant (estimate = 3.93; *p* = 0.04). This means that higher scores in the scale *Active Stress Coping* were associated with higher situational coping in daily life. The effects of the other coping styles on situational coping did not reach statistical significance.

**Table 4:** Results of the multilevel model investigating the effects of coping styles on situational coping in the total sample.

Parameter	Estimate (SE)	Statistics
Intercept	66.75 (1.76)	*t*(113.81) = 38.01; *p* < 0.001
Coping strategy scale: positive thinking	4.11 (1.89)	*t*(112.59) = 2.18; *p* = 0.03
Coping strategy scale: active stress coping	3.93 (1.91)	*t*(116.94) = 2.06; *p* = 0.04
Coping strategy scale: social support	1.15 (1.90)	*t*(114.45) = 0.60; *p* = 0.55
Coping strategy scale: support in faith	−1.77 (1.88)	*t*(111.88) = −0.94 *p* = 0.35
Coping strategy scale: alcohol and cigarette consumption	−1.00 (1.79)	*t*(113.21) = −0.56; *p* = 0.58

*SE, Standard Error, the coping strategy scales were z-standardized*.

### Results for RQ 1a

Table 5 shows the results of the multilevel model testing the effects of coping styles on situational coping for women. The effect of the SCI scale *Social Support* on situational coping was positive and significant (estimate = 6.30; *p* = 0.046). This means that higher scores in the scale *Social Support* were associated with higher situational coping for women.

**Table 5:** Results of the multilevel model investigating the effects of coping styles on situational coping for women.

Parameter	Estimate (SE)	Statistics
Intercept	61.59 (2.53)	*t*(63.53) = 24.39; *p* < 0.001
Coping strategy scale: positive thinking	4.14 (2.52)	*t*(63.26) = 1.65; *p* = 0.11
Coping strategy scale: active stress coping	0.82 (2.45)	*t*(64.02) = 0.34; *p* = 0.74
Coping strategy scale: social support	6.30 (3.10)	*t*(62.11) = 2.03; *P* = 0.046
Coping strategy scale: support in faith	−1.01 (2.45)	*t*(62.19) = −0.41; *p* = 0.68
Coping strategy scale: alcohol and cigarette consumption	−0.57 (2.50)	*t*(61.78) = −0.23; *p* = 0.82

*SE, Standard Error, the coping strategy scales were z-standardized*.

### Results for RQ 1b

Table 6 shows the results of the multilevel model testing the effects of coping styles on situational coping for men. The effect of the SCI scale *Active Stress Coping* was positive and significant (estimate = 8.94; *p* = 0.001). This means that higher scores in the scale *Active Stress Coping* were associated with higher situational coping in daily life for men.

**Table 6:** Results of the multilevel model investigating the effects of coping styles on situational coping for men.

Parameter	Estimate (SE)	Statistics
Intercept	73.54 (2.31)	*t*(46.31) = 31.84; *p* < 0.001
Coping strategy scale: positive thinking	0.95 (2.55)	*t*(47.42) = 0.37; *p* = 0.71
Coping strategy scale: active stress coping	8.94 (2.59)	*t*(54.82) = 3.45; *p* = 0.001
Coping strategy scale: social support	0.79 (2.14)	*t*(47.74) = 0.37; *p* = 0.71
Coping strategy scale: support in faith	−1.16 (2.59)	*t*(49.73) = −0.45; *p* = 0.66
Coping strategy scale: alcohol and cigarette consumption	−1.16 (2.15)	*t*(50.28) = −0.54; *p* = 0.59

*SE, Standard Error, the coping strategy scales were z-standardized*.

### Results for RQ 1c

Table 7 shows the results of the multilevel model testing the interaction effects of gender and coping styles on situational coping. The effect of main effect of *SS* was significant (*p* = 0.028). The estimate was positive (β = 2.35). As women were coded as 0 in this model, the main effect for *SS* means that higher scores in the SCI subscale *SS* were associated with higher situational coping for women. The two-way interaction effect between *AS* and sex was significant (*p* = 0.041). The estimate was positive (β = 3.62). As men were coded as 1 in this model, the interaction effect of AS and gender means that higher scores in the SCI subscale *AS* were associated with higher situational coping for men in comparison with women.

**Table 7:** Fixed effects of the linear multilevel model testing the interaction effect of coping styles and gender on situational coping.

Parameter	Estimate	SE	df	*T*	Value of *p*	95% CI
Intercept	8.51	19.53	105.42	0.436	0.664	[−30.22, 47.23]
PT	1.77	0.97	108.13	1.814	0.072	[−0.16, 3.70]
AS	0.44	1.02	109.43	0.434	0.665	[−1.58, 2.46]
SS	2.35	1.05	105.93	2.232	0.028*	[0.263, 4.44]
SF	−0.46	0.96	106.11	−0.478	0.633	[−2.37, 1.45]
AC	−0.21	0.86	105.34	−0.240	0.811	[−1.92, 1.50]
gender	15.57	29.00	118.71	0.537	0.592	[−41.86, 73.00]
PT * gender	−1.34	1.64	111.69	−0.818	0.415	[−4.58, 1.90]
AS * gender	3.62	1.75	123.24	2.069	0.041*	[0.16, 7.09]
SS * gender	−2.06	1.43	110.00	−1.434	0.154	[−4.90, 0.79]
SF * gender	−0.04	1.66	113.94	−0.027	0.979	[−3.34, 3.25]
AC * gender	−0.26	1.31	113.32	−0.198	0.843	[−2.85, 2.34]

*Women were coded as 0 and men were coded as 1 in this multilevel model. CI, confidence interval; PT, positive thinking; AS, active stress coping; SS, social Support; SF, support in faith; AC, increased alcohol and cigarette consumption*.

* *p < 0.05*.

### Results for RQ 2

The results of the t-tests are depicted in Table 8. Women scored significantly higher in the SCI scale *Social Support* than men (*p* = 0.007). As the Levene test revealed lacking heteroscedasticity for this predictor, the corrected degrees of freedom are reported. The effect size was medium (Hedge’s *g* = 0.56). This result was still significant after Bonferroni correction (five tests: 0.05/5 results in *p* = 0.01). No gender-differences between women and men emerged for any of the other four coping styles.

**Table 8:** Differences in coping styles between women and men.

	Women		Men		*T (df)*			Value of *p*	*Hedge’s g*
	*M*	*SD*	*M*	*SD*
PT	11.45	2.33	12.06	2.34	−1.39 (110)	0.17	−0.26
AS	11.65	2.23	11.45	2.14	0.48 (110)	0.64	0.09
SS	14.14	2.07	12.68	3.16	2.77 (73.77)	0.007	0.56
SF	7.27	2.31	6.45	2.23	1.87 (110)	0.07	0.35
AC	6.52	2.51	6.85	2.73	−0.66 (110)	0.51	−0.12

*PT, positive thinking; AS, active stress coping; SS, social support; SF, support in faith; AC, alcohol and cigarette consumption*.

## Discussion

### Principal Findings

This EMA study investigated the effects of five coping styles (positive thinking, active stress coping, social support, support in faith, and alcohol and cigarette consumption) on situational coping in daily life. In the total sample, the two SCI scales *Positive Thinking* and *Active Stress Coping* had significant positive effects on situational coping in daily life. This result was in line with our hypothesis assuming differences between the coping styles in their effect on situational coping. In addition, the result corresponds with principal findings about the SCI coping scales from retrospective assessments, which showed that both *Positive Thinking* and *Active Stress Coping*, as well as *Social Support* were negatively correlated with stress symptoms (Satow, 2012). In contrast to these results, however, the effect of *Social Support* on situational coping overall did not reach statistical significance in our total sample.

When investigating gender-specific effects of the coping styles on situational coping, social support was associated with higher situational coping for women, whereas active stress coping was associated with higher situational coping for men. These results are again in line with our hypotheses, assuming that the coping styles differ in their effect on situational coping in women and men. In contrast to the results for the total sample, positive thinking did not have a significant impact on situational coping for women/men alone. It is possible that this effect would have reached statistical significance if the size of the subsample of women/men would match the one of the total sample.

The interaction effects of the coping styles and gender show that active stress coping was more strongly associated with situational coping for men than for women. This confirms the weight of active stress coping in RQ1b. Social support was significantly associated with situational coping for women as seen in the results of RQ1a and RQ1c, but this association was not significantly stronger for women than for men as the interaction between active stress coping and gender did not reach significance.

Women in our sample had higher scores on the SCI scale *social support* than men with a medium effect size, indicating that women are more likely than men to use social support as a coping strategy. This confirms our hypothesis regarding gender-differences in coping styles and is in line with the existing literature (Tamres et al., 2002;Brougham et al., 2009; Steinert and Haesner, 2019). The result that no other coping strategy differed between women and men is in contrast to the literature. A meta-analysis on gender-differences in coping behaviors suggested that especially women use active stress coping styles (Tamres et al., 2002). More recent studies also support these findings (Brougham et al., 2009; Steinert and Haesner, 2019). On the other hand, some earlier studies also suggest that men use active coping styles more often. As mentioned above, for example, Stone and Neale (1984) reported that men in their sample used direct action as a coping strategy more often than women, whereas the women used coping styles such as seeking social support more often, which is in line with our results. These studies, however, assessed the specific coping styles used in stressful situations while we assessed the individual coping styles of our participants, which could account for some of the differences between our results and the existing literature.

### Limitations and Strengths

There are various limitations of this study. One of these is the rather low internal validity due to the non-experimental design and the assessments being made in everyday life settings, which made it impossible to control for potential confounding variables. Another limiting factor is that the sample was not recruited randomly, but rather at universities and in the social networks of the three student recruiters. The sample is, therefore, not representative for the general population. To maintain maximum anonymity, possible relationships between participants and recruiters were not assessed. It will be possible to have a more representative sample when TrackYourStress is made available in the app stores. Moreover, it is important to note that we only once assessed the tendency to use certain coping styles in daily life at the start of the study. The specific coping strategies applied by the participants in specific situations were not assessed.. Assessing coping multiple times a day at random time points would increase the obtained information. Future studies could include incentives to more strongly encourage participants to provide multiple assessments per day. Another limitation is the potential bias in ratings of situational coping due to these ratings being made at self-chosen time-points. It is possible that participants only filled out the questionnaires in moments of high perceived situational coping or in moments without any additional stressors. Aside from these limitations, this study also holds several strengths. Firstly, the ecological validity of the study is high as the assessment method of EMA allows the generalization of the results to the context of everyday life. Secondly, we used a reliable and valid questionnaire to assess individual coping styles.

Future studies with TrackYourStress should focus on controlling possible confounding variables to increase internal validity, by more specifically assessing the stressful situation and context variables. A more holistic approach for future research could be to create an index for the breadth of coping strategies used in order to investigate whether participants with more flexibility in coping strategies show better stress response and coping. Some studies have shown that a greater variety of coping strategies can lead to a better coping outcome than focusing on one specific strategy (O’Connell et al., 2007; Witkiewitz et al., 2018). When accounting for the variety of coping strategies used, it would be possible to move away from the traditional view of coping strategies as either adaptive or maladaptive and instead focus on a strategy-situation fit as proposed by Haines et al. (2016). Assessing any pre-existing conditions that could affect the experienced stress levels and situational coping of the participants, such as personality factors as well as health related variables, should also be considered in the future. Implementing a semi-random or stratified random sampling protocol with multiple assessments per day, made during periods of situational coping, would also be interesting in order to investigate daily fluctuations in situational coping. By defining strata or time intervals in a day, during which assessments are scheduled at random, the assessments are evenly sampled throughout the day (Shiffman et al., 2008). In addition to the subjective assessment of situational coping, future studies could also include the change in stress levels after a coping attempt as a coping outcome and analyze the overlap between these two outcomes.

### Implications

The findings from the present study could be relevant for interventions aimed at increasing adaptive coping. Our findings suggest that active stress coping could be associated with better situational coping for men than for women. Gender differences for the other coping styles, however, should be interpreted with caution, as these interaction effects did not reach significance. Nevertheless, the meaningfulness of gender-specific health promotion is also supported by findings from other studies. For instance, a study with German university students showed that drug-taking behaviors were more common in male students, whereas preventive behaviors such as healthy nutrition were more common in female students (Stock et al., 2019). Furthermore, the female students had a stronger interest in most health promotion programs than male students and this interest could be predicted by alcohol in male students and psychosocial stress in female students. It would be interesting to investigate whether gender-specific interventions that take such differences into account can lead to better results than generalized interventions. In this regard, it would be relevant to assess which specific coping styles women or men use in specific daily life situations. However, although women and men can significantly differ from one another on average, there can still be some overlap in coping behaviors. Therefore, a more sensible approach altogether might be to develop coping interventions tailored to individual coping behaviors. It would even be possible to combine TrackYourStress with an Ecological Momentary Intervention (EMI) to help women or men in coping for example by mindful walking (Pryss et al., 2018) or functional relaxation (Lahmann et al., 2017). A promising approach is to use so-called just-in-time interventions, which might support individuals when significant changes in stress levels or situational coping are detected (Clarke et al., 2017). A recent study showed that participants’ previous stress ratings were most successful at predicting future stress ratings on a larger scale and that environmental factors were most successful at predicting future stress ratings on a more individual level (Pryss et al., 2019a). To design personalized just-in-time coping-interventions in daily life to reduce stress-related health risks, it would therefore be advisable to have combined assessments of personal and environmental factors.

## Conclusion

The coping styles *Positive Thinking* and *Active Stress Coping* were associated with better situational coping in daily life settings. While social support was a coping strategy predicting enhanced situational coping for women, active stress coping was associated with better situational coping for men. Moderation analyses revealed that SS was not significantly more important for situational coping for women than for men, but AS was significantly more important for men than for gender. These results suggest that different coping styles could be more effective in daily life for women than for men, which should be followed up in future EMA studies and be considered in the development of interventions aimed at reducing stress consequences through coping.

## Data Availability Statement

The raw data supporting the conclusions of this article will be made available by the authors, without undue reservation.

## Ethics Statement

Ethical review and approval was not required for the study on human participants in accordance with the local legislation and institutional requirements. The patients/participants provided their written informed consent to participate in this study.

## Author Contributions

TO contributed to writing—original draft, writing—review and editing, and data analysis and interpretation. CV, DJ, and MR contributed to study design, data collection, and writing—review and editing. RP, CP, UN, and AF contributed to writing—review and editing. JS and TP contributed to data collection, data analysis and interpretation, study design, and writing—review and editing. FH and JH contributed to data collection and writing—review and editing. All authors contributed to the article and approved the submitted version.

## Conflict of Interest

The authors declare that the research was conducted in the absence of any commercial or financial relationships that could be construed as a potential conflict of interest.

## Publisher’s Note

All claims expressed in this article are solely those of the authors and do not necessarily represent those of their affiliated organizations, or those of the publisher, the editors and the reviewers. Any product that may be evaluated in this article, or claim that may be made by its manufacturer, is not guaranteed or endorsed by the publisher.
